# The removal of multiplicative, systematic bias allows integration of breast cancer gene expression datasets – improving meta-analysis and prediction of prognosis

**DOI:** 10.1186/1755-8794-1-42

**Published:** 2008-09-21

**Authors:** Andrew H Sims, Graeme J Smethurst, Yvonne Hey, Michal J Okoniewski, Stuart D Pepper, Anthony Howell, Crispin J Miller, Robert B Clarke

**Affiliations:** 1 Applied Bioinformatics of Cancer Research Group, Breakthrough Research Unit, Edinburgh Cancer Research Centre, Western General Hospital, Crewe Road South, Edinburgh, EH4 2XR, UK; 2 Breast Biology Group, School of Cancer and Imaging Sciences, University of Manchester, UK; 3 Cancer Research UK Applied Computational Biology and Bioinformatics Group; 4 Cancer Research UK Affymetrix Service, Paterson Institute for Cancer Research, Wilmslow Road, Manchester M20 4BX, UK; 5 Functional Genomics Center, UNI ETH Zurich, Winterthurerstrasse 190, CH-8057 Zurich, Switzerland

## Abstract

**Background:**

The number of gene expression studies in the public domain is rapidly increasing, representing a highly valuable resource. However, dataset-specific bias precludes meta-analysis at the raw transcript level, even when the RNA is from comparable sources and has been processed on the same microarray platform using similar protocols. Here, we demonstrate, using Affymetrix data, that much of this bias can be removed, allowing multiple datasets to be legitimately combined for meaningful meta-analyses.

**Results:**

A series of validation datasets comparing breast cancer and normal breast cell lines (MCF7 and MCF10A) were generated to examine the variability between datasets generated using different amounts of starting RNA, alternative protocols, different generations of Affymetrix GeneChip or scanning hardware. We demonstrate that systematic, multiplicative biases are introduced at the RNA, hybridization and image-capture stages of a microarray experiment. Simple batch mean-centering was found to significantly reduce the level of inter-experimental variation, allowing raw transcript levels to be compared across datasets with confidence. By accounting for dataset-specific bias, we were able to assemble the largest gene expression dataset of primary breast tumours to-date (1107), from six previously published studies. Using this meta-dataset, we demonstrate that combining greater numbers of datasets or tumours leads to a greater overlap in differentially expressed genes and more accurate prognostic predictions. However, this is highly dependent upon the composition of the datasets and patient characteristics.

**Conclusion:**

Multiplicative, systematic biases are introduced at many stages of microarray experiments. When these are reconciled, raw data can be directly integrated from different gene expression datasets leading to new biological findings with increased statistical power.

## Background

Successful microarray experiments are reliant on sufficient care being taken to minimize and account for experimental variability. Formalization and control of all stages of the experimental pipeline is now routine, and the need to associate experiments with detailed descriptions of protocols and techniques is now widely accepted [1]. However, despite these efforts, it is still not possible to account for all potential sources of variation, and even identical experiments performed at different sites have been shown to produce significantly different results [2]. This makes it difficult to routinely compare gene expression data generated from different experiments, even when using samples from comparable sources that have been processed on the same microarray platform using similar protocols.

Issues of experimental reproducibility have become increasingly important with the advent of microarray databases and repositories (e.g. ArrayExpress [1], GEO [3]), given the potential they offer for cross-experimental comparison and data mining. Even if it is possible to successfully control inter-experiment variation to a point where this might be possible, rapid developments in both the hybridization protocols and in the arrays themselves have also led to major improvements in the technology. Lower requirements for the amount of starting RNA have enabled gene expression profiling to be combined with cell sorting methods or laser capture microdissection, while increases in the number of features represented on the arrays have resulted in progressively more detailed coverage of the transcriptome. Since each advance in technology leads to genuine improvements, there is a strong incentive to use the latest arrays and protocols whenever possible. This is however, tempered by a lack of backward compatibility between datasets produced using different array and protocol versions, and any decision to move to a newer (better) iteration of the technology must be made with an appreciation of the difficulty in maintaining compatibility with previous studies.

In this study we first demonstrate, using an extended series of validation data, that Affymetrix datasets cannot in general be compared at the raw expression level due to systematic, multiplicative biases. Secondly, we show that simple batch mean-centering can significantly reduce the level of inter-experimental variation and that this allows raw transcript levels to be compared across datasets. The approach is then applied to a series of published breast cancer studies and we show that the integrated datasets possess increased statistical power and improved prognostic ability, compared to the individual datasets alone.

## Results

### Systematic bias in microarray data

**Figure 1:**
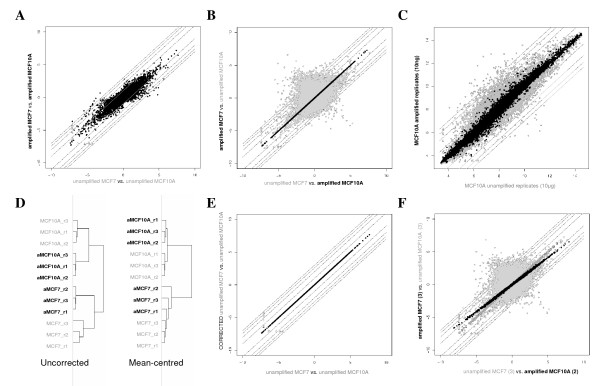
**Comparison of Affymetrix gene expression data generated using amplified and unamplified protocols**. A, Comparing fold changes *between *unamplified and amplified datasets demonstrates reasonable correlation. B, Comparing fold changes *across *datasets (unamplified MCF7 with amplified MCF10A and vice versa) is clearly impractical (grey spots), however following mean batch-centering there is excellent correlation across the datasets (black spots). C, Comparison of mean raw expression levels for amplified and unamplified MCF10A replicates before (grey) and after mean batch-centering (black). D, Pearson clustering of the GeneChips representing the same cell lines is tighter following mean-centering. E, Mean-centering has no effect on fold changes between datasets. F, Mean-centering of unbalanced datasets (duplicate rather than triplicate amplified MCF10A) results in a distortion of the comparison (black spots), however this is rectified with weighted mean-centering (open dark grey spots), both methods show a dramatic improvement over uncorrected data (light grey spots).

All validation datasets (consisting of six GeneChips each) were produced by hybridizing triplicate RNA samples from a breast cancer cell line (MCF7) and an immortalised normal breast cell line (MCF10A) using a variety of different array types and sample preparation protocols. In all cases, the aim of the validation study was to assess the correspondence between the sets of differentially expressed probesets (or transcripts) identified when using different versions of the same underlying technology. In the hypothetical situation when all datasets yield identical results, the same set of differentially expressed probesets would be identified, irrespective of the array type or protocol used to process the data; we considered how close the data approaches this ideal. For example, a comparison of the fold changes *between *datasets (MCF7-amplified/MCF10A-amplified vs. MCF7-unamplified/MCF10A-unamplified) generated using Affymetrix's small sample protocol and Affymetrix's standard protocol yields good, but not perfect correspondence (Fig 1A, Table 1). However, when fold changes are calculated *across *datasets (MCF7-amplified/MCF10A-unamplified vs. MCF7-unamplified/MCF10A-amplified) correspondence falls dramatically (Fig. 1B grey dots, Table 1).

**Table 1:** Summary of the effect of mean batch-centering on data generated from different experiments.

		Within two-fold consistent (%)				SAM Common, top 1000			
		
Data from different experiments	Probesets	Between datasets		Across datasets		Between datasets		Across datasets	
		
		Before	After	Before	After	Before	After	Before	After
Amplified 10 ng and unamplified 10 μg protocols, RMA, U133A	22283	20645 (92%) (Fig 1A)	20645 (92%) (Fig 1A)	13221 (59%) (Fig 1B)	22283 (100%) (Fig 1B)	522 (0.031) (0.032)	522 (0.031) (0.032)	251 (0.023) (0.037)	594 (0.025) (0.035)
U133A and plus 2.0 arrays common, MAS5 present = 4/6 chips	11198	10641 (95%)	10669 (95%)	2675 (24%)	11170 (100%)	493 (0.037) (0.036)	493 (0.037) (0.036)	112 (0.026) (0.027)	954 (0.041) (0.040)
Exon v U133 plus 2 (consensus mapping) Plier and MAS5	44280	37255 (84%)	37255 (84%)	9485 (21%)	44280 (100%)	528 (0.067) (0.060)	528 (0.067) (0.060)	569 (0.024) (0.020)	847 (0.110) (0.089)
Exon v U133 plus 2 (SIF mapping) Plier and MAS5	13730	12028 (88%)	12028 (88%)	3626 (26%)	13730 (100%)	524 (0.068) (0.060)	524 (0.068) (0.060)	731 (0.026) (0.026)	916 (0.569) (0.370)
Standard 10 μg and revised 2 μg protocols, RMA, U133A	22283	21303 (96%)	21303 (96%)	19779 (89%)	22283 (100%)	688 (0.035) (0.050)	688 (0.035) (0.050)	618 (0.039) (0.044)	901 (0.044) (0.042)
GeneChip 3000 and GeneArray 2500 scanners, RMA, U133A	22283	22276 (100%)	22276 (100%)	22282 (100%)	22283 (100%)	883 (0) (0)	883 (0) (0)	872 (0) (0)	879 (0) (0)
NuGEN 10 ng and EpiStem 2 ng amplification, RMA, U133 plus 2	54675	49308 (90%)	49308 (90%)	28276 (52%)	54675 (100%)	530 (0.060) (0.066)	530 (0.060) (0.066)	364 (0.035) (0.034)	743 (0.070) (0.069)

Sets of differentially expressed probesets comparing MCF7 and MCF10A replicates were identified for each experiment, before and after mean batch-centering. Comparisons between and across different validation experiments were performed. The number (%) of probesets with less than 2-fold deviation in the fold change found for each comparison is reported in the table. SAM Common: for each column two different pairwise comparisons using SAM were performed, and the top 1000 probesets identified for each comparison. The number reported is the intersection between the two sets. Before: comparison was performed prior to mean batch-centering. After: comparison was performed following mean batch-centering. Values in brackets are the FDR for each top 1000 probesets. See Additional File 1 for plots.

Batch mean-centering (see methods) of the amplified and unamplified datasets was found to dramatically increase the correspondence comparison *across *the datasets, with 100% of probesets having fold changes within two-fold between experimental branches (Fig. 1B black dots, Table 1). Similarly, when significance analysis of microarrays (SAM) was used to identify lists of probesets with statistically significant changes between the same replicate groupings used to generate Fig. 1, the intersection between sets was also found to be greater following mean batch correction (Table 1) and Pearson correlation of raw intensities was also found to increase (Figs. 1C, 1D and Additional File 1). It is notable that while correction improves fold-change correspondence *across *protocols, fold changes *between *datasets are preserved (Fig. 1E, Table 1).

The same approach was applied to a variety of other validation datasets that were designed to investigate the effect of using different generations of Affymetrix GeneChips, alternative protocols and scanning hardware (Table 1, Additional File 1). A systematic bias was found to be present in all datasets, and correspondence improved following mean-centering in all cases (median centering also performed similarly; data not shown). These results demonstrate that systematic, multiplicative bias is a widespread property within Affymetrix array data, and mean-centering was found to lead to improvements irrespective of the summarisation method used (RMA or MAS5 [4]) to process the data or when using alternative Chip Description Files (CDFs) to group probes according to Unigene cluster [5] (Additional File 2).

### Integration of published breast tumour datasets

**Figure 2:**
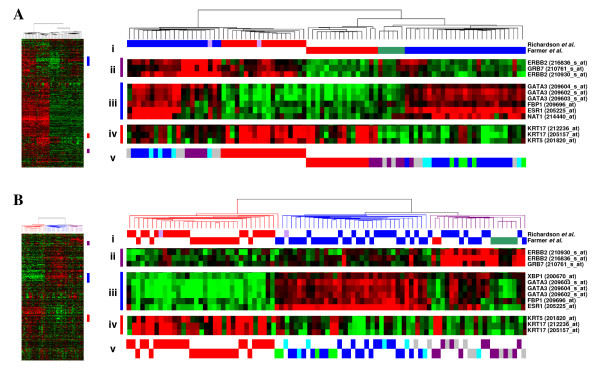
**Comparison of breast tumour gene expression profiles generated by two published studies**. The Farmer *et al. *study used U133A GeneChips with RNA amplification, whereas the Richardson *et al. *study used U133 plus 2.0 arrays and the standard labeling protocol. A, Before mean batch-centering. B, After mean batch-centering. Hierarchical clustering of tumours based upon 640 probesets representing Sorlie *et al. *[8] 'intrinsic' genes. Thumbnails show all 640 probesets. i) Tumours classified by Richardson *et al. *[10] red = basal-like, blue = non-basal like, pink = BRCA1; tumours classified by Farmer *et al. *[11] red = basal, blue = luminal, green = apocrine. Clusters of genes associated with the 'Sorlie subtypes' are highlighted as follows; ii) ERBB2 gene cluster, iii) luminal A gene cluster, iv) basal gene cluster. v) Centroid prediction was used to assign the tumours to the five Norway/Stanford subtypes – basal (red), luminal A (dark blue), luminal B (light blue), ERBB2 (purple) and normal-like (green), unassigned (grey).

Breast tumours have been classified into five molecular subtypes; basal, luminal A, luminal B, ERBB2 and normal-like by identifying a set of genes with significantly greater variation in expression between different tumours than between paired samples from the same tumour [6-8]. Since members of this set appear to define properties 'intrinsic' to each subtype, the authors referred to the genes as an 'intrinsic gene set'. 640 Affymetrix probesets representing the 534 'intrinsic gene set' from [13] were identified using NetAffx [9]. These probesets were used to cluster MAS5 normalised expression data from two published Affymetrix gene expression studies [10,11] with similar numbers and composition of tumours. Despite using similar starting material (primary tumours) and the same microarray platform, when combined the two datasets formed two distinct, independent clusters representing the two datasets (Fig. 2A), suggesting a dataset-specific systematic bias as observed with the validation datasets described above. Although clusters of known luminal and basal-specific genes show similar patterns of differential expression in each of the two datasets (Figure 2A iii, iv), the majority of the probesets representing the full 'intrinsic gene set' show greater differences in expression between the two studies than between the different classes of tumours. Following mean-centering as before, the 'basal-like tumours' from the Richardson *et al. *[10] dataset were found to cluster alongside the 'basal tumours' from Farmer *et al. *[11], and the 'non-basal tumours' with the 'luminal tumours' (Fig. 2B). A third cluster of tumours was identified with high expression of the ERBB2 cluster of probesets. This cluster contained all of the molecular apocrine tumours, plus a mixture of basal/basal-like and luminal/non-basal-like tumours. Using centroid prediction [12] as described previously, the tumours from both datasets were assigned to the five Norway/Stanford subtypes (basal, luminal A, luminal B, ERBB2 and normal-like [6-8]), one of the tumours in this third cluster was assigned to the luminal B subtype, thirteen to the ERBB2 subtype and 9 could not clearly be assigned to a subtype (Fig 2B v).

The greatest single difference between molecular subtypes has repeatedly been demonstrated to be between estrogen receptor-positive (ER+) luminal tumours and ER-negative basal tumours [6-8,13,14]. SAM analysis was used to identify probesets differentially expressed between the basal/basal-like and non basal-like/luminal subtypes using the combined data from both sets of samples. It was only following mean-centering that probesets were identified that represent genes that are known to characterize the differences between these subtypes (Additional File 3), including the fundamental estrogen receptor alpha and GATA binding protein 3, which maintains differentiation into the luminal cell fate in the mammary gland [15]. In addition, following mean-centering, a greater number of statistically significant probesets were found to be differentially expressed between the tumour subtypes than were found between the two initial sets of samples (Additional File 4).

**Figure 3:**
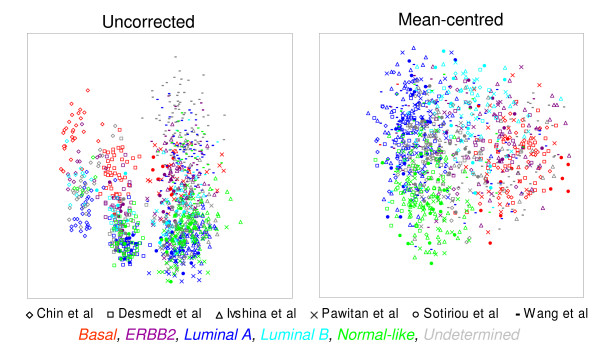
**Dataset-specific bias in published Affymetrix breast cancer studies**. Multidimensional scaling for all common probesets (22,215) for 1107 breast tumours from six published studies [16-21] on U133A, U133AA and U133 plus 2.0 GeneChips. Tumours from different datasets are distinguished by symbol. Tumours assigned to one of the five Sorlie *et al. *subtypes by centroid prediction are discriminated by colours. With uncorrected data the tumours cluster by study, following mean-centering the tumours cluster by molecular subtype.

These results encouraged us to apply the approach to integrate six previously published datasets [16-21] (Table 2) of primary breast cancer tumours processed on Affymetrix U133A, U133AA or plus 2.0 GeneChips. Multidimensional scaling of 1107 tumours based upon the expression of all common probesets between the three arrays (22,215) demonstrated that global gene expression is highly influenced by dataset, with tumours clustering by study (Fig. 3A), again suggesting a systematic, dataset-specific bias. However, following mean-centering, the tumours appear to cluster by breast cancer subtype (assigned using centroid prediction [12]), regardless of the dataset from which they were generated (Fig. 3B).

**Table 2:** Published breast cancer datasets used in this study.

**Datasets**	**No. Tumours**	**Array express/GEO ID**	**GeneChip**	**ER+**	**Age**	**Tumour Size (cm)**	**FU (years)**	**Reference**
Chin *et al. *2006	114	E-TABM	U133AA	67%	51	2.3	6.1	[16]
Desmedt *et al. *2007	198	GSE7390	U133A	68%	47	2.0	13.6	[17]
Farmer *et al *2005	49	GSE1561	U133A	58%	-	-	-	[11]
Ivshina *et al. *2006	249	GSE4922	U133A	85%	63	2.0	9.9	[18]
Loi *et al. *2007	119, 87	GSE6532	U133A, U133 plus2.0	100%	65, 62	2.4, 2.1	5.2, 11.4	[32]
Minn *et al. *2007	58	GSE5327	U133A	0%	-	-	7.2	[33]
Pawitan *et al. *2005	159	GSE1456	U133A	83%	58^$^	2.2^$^	7.1	[19]
Richardson et al.	40	GSE3744	U133 plus2.0	38%	-	-	-	[10]
Sotiriou *et al. *2006	101*	GSE2990	U133A	71%	60	2.0	5.8	[20]
Wang *et al. *2005	286	GSE2034	U133A	73%	52	-	7.2	[52]

Continuous variables (age, size and follow up) are given as median values,  except where indicated ^$^ the mean was given. The follow up (FU) endpoints  for the datasets Loi et al, Pawitan et al. and Sotoriou et al were  recurrence-free survival, for datasets Desmedt et al. and Ivshina et al. it  was disease-free survival and for datasets Minn et al. and Wang et al. it  was distant metastasis-free survival. *The full dataset of Sotiriou et al.  includes 189 tumours, but 88 of the Uppsala tumours are included in dataset  Ivshina et al.

**Figure 4:**
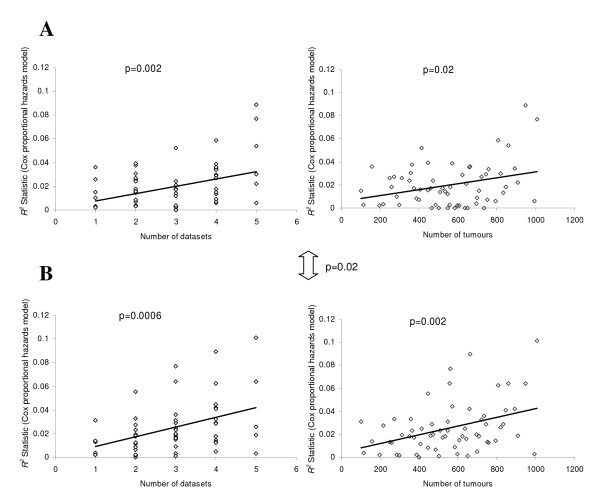
**Combining datasets or tumours and mean-centering significantly increases prognostic prediction**. A, Before mean batch-centering. B, After mean batch-centering. The *R*^2 ^statistic (Cox proportional hazards model) is an assessment of the performance of the predictor generated using each combination of training datasets and the remaining test datasets, generated using supervised principal components analysis. Median values are used where a training dataset was used to assess more than one test dataset (up to 5). *R*^2 ^and *p-value *results for all possible combinations of training datasets and test datasets (1016) are given in the matrix in Additional File 6.

A growing body of evidence has accumulated, supporting the notion that gene expression profiling of primary breast tumours can be used to stratify patients by subtype and the likelihood of disease progression (reviewed in [22]). The approaches have included both unsupervised (intrinsic gene set [6-8]) and supervised (genes associated with patient follow up [17,21,23]) methods, along with studies of cancer-associated pathways or tumor characteristics [24-27], in all cases these signatures appear to predict recurrence, despite the lack of overlap in their respective profiles or signatures [28]. In order to establish whether integrating multiple datasets can improve prognostic prediction, the six published datasets (described above) were used individually or in combination as 'training sets' for supervised principal components analysis [29,30]. This method has been shown to produce more accurate predictions than several competing methods on both simulated and real microarray datasets [29,30]. Using the Superpc [30] package for R [31], a Cox proportional hazards model was fitted to each predictor (generated for all combinations of one to five datasets used as the 'training set') and cross validation curves were plotted to determine the optimum threshold for the predictor of survival as described previously [30]. The 1^st ^supervised principal component was found to be the most significant in the vast majority of cases, which is consistent with the hypothesis that recurrence is an inherent property of primary tumour gene expression (examples of cross-validation and survival curves are shown in Additional File 5). The remaining dataset(s) were used as a test set for each predictor and an *R*^2 ^statistic was computed to assess the performance. Combining greater numbers of datasets or tumours significantly improves prediction of prognosis based upon gene expression data (Fig. 4). Mean-centering of the datasets significantly increased the correlation between the supervised principal components and clinical follow up, therefore improving prognostic performance. It is clear that the predictive power of some combinations of training and test sets is more reliable than others. Although only a limited number of patient and tumour characteristics were available (Table 2), it seems that the most accurate predictions are achieved for test datasets that have characteristics most similar to those of the individual or combined training dataset. *R*^2 ^statistic and p-values (log rank) for all possible combinations of training datasets and test datasets are given in Additional File 6.

### Dataset composition

We investigated the effect of altering the composition of luminal (ER+) and basal (ER-) tumours from the two published datasets of Farmer *et al. *[11] and Richardson *et al. *[10] compared above. Unbalancing the composition of the datasets from a 1:1 ratio of basal to luminal tumours to a 2:1 or 5:1 ratio of tumours reduced correspondence *between *datasets and caused a distortion *across *datasets (Additional File 7). Similar results were also observed with the MCF7/MCF10A datasets described above (Fig 1F, Table 3). Weighted-mean-centering for ER status removed the distortion but also reduced correspondence for the 2:1 ratio of luminal tumours, and increased correspondence in the 5:1 ratio luminal to basal comparison, at the expense of high false discovery rates (Table 3). An extreme example of the effect of dataset composition was seen when looking at the expression level of the estrogen receptor in homogeneous datasets from Loi *et al. *[32] and Minn *et al. *[33] composed wholly of ER+ or ER- tumours. Following mean-centering it appears that these datasets contain a mixture of ER+ and ER- tumours (Additional File 8A). Replacing any of the six heterogenous datasets above (containing 67–85% ER+ tumours) with homogeneous datasets (containing only ER+ or ER- tumours) showed a dramatic reduction in the correlation between dataset or tumour number and prediction of recurrence (Additional File 8B). Using weighted mean-centering to account for the differences in the composition of ER+ tumours in five out of the six datasets (individual ER status by immunohistochemistry for tumours in the Pawitan *et al. *dataset was not available) did not significantly improve prognostic performance over mean-centering alone (Additional File 9).

**Table 3:** Effect of dataset composition on differential gene expression.

	SAM Common, top 1000							
Uneven comparisons	Between datasets				Across datasets			
	
	UC	MC	wMC	DWD	UC	MC	wMC	DWD

Unamplified MCF7 (3) v MCF10A (3) Amplified MCF7 (3) v MCF10A (3)	522 (0.031) (0.032)	522 (0.031) (0.032)	-	427 (0.029) (0.028)	251 (0.023) (0.037)	594 (0.025) (0.035)	-	447 (0.023) (0.032)
Unamplified MCF7 (3) v MCF10A (3) Amplified MCF7 (3) v MCF10A (2)	495 (0.031) (0.036)	495 (0.031) (0.036)	495 (0.031) (0.036)	469 (0.03) (0.031)	232 (0.026) (0.035)	600 (0.024) (0.037)	597 (0.026) (0.040)	550 (0.028) (0.0)
								
Richardson *et al. *Non-basal (12) v basal (12) Farmer *et al. *Luminal A (12) v basal (12)	394 (0.003) (0.019)	394 (0.003) (0.019)	-	389 (0.003) (0.019)	368 (0.001) (0.019)	708 (0.047) (0.02)	-	695 (0.046) (0.014)
Richardson *et al. *Non-basal (7) v basal (19) Farmer *et al. *Luminal A (15) v basal (14)	380 (0.019) (0.001)	380 (0.019) (0.001)	380 (0.019) (0.001)	373 (0.001) (0.017)	346 (0) (0)	725 (0.003) (0.078)	608 (0.002) (0.038)	658 (0.005) (0.021)
Richardson *et al. *Non-basal (3) v basal (19) Farmer *et al. *Luminal A (15) v basal (3)	283 (0.1) (0.194)	283 (0.1) (0.194)	283 (0.1) (0.194)	258 (0.195) (0.099)	290 (0) (0.027)	480 (0.093) (0.9)	684 (0.001) (0.789)	506 (0.112) (0.9)

Sets of differentially expressed probesets comparing MCF7 and MCF10A replicates or basal/basal-like and luminal/nonbasal-like tumours were identified for each experiment, before and after mean batch-centering, comparisons both between and across datasets were performed. SAM Common: for each column two different pairwise comparisons using SAM were performed, and the top 1000 probesets identified for each comparison. The number reported is the intersection between the two sets. Before: comparison was performed prior to mean batch-centering. After: comparison was performed following mean batch-centering. Values in brackets are the FDR for each top 1000 probesets. Weighted mean-centering for datasets with even numbers of samples are not shown as the values are identical to mean-centering. UC = uncorrected, MC batch mean-centered, wMC = weighted mean-centered, DWD = distance-weighted discrimination.

The mean-centering approach was compared with a previously described method for integrating breast cancer tumour microarray data generated on different platforms, using a distance weighted discrimination (DWD) method to adjust for systematic microarray data biases [34]. For both the validation datasets and the published datasets, mean-centering out-performed DWD (Table 3 and Additional File 10).

### Most variable genes

**Figure 5:**
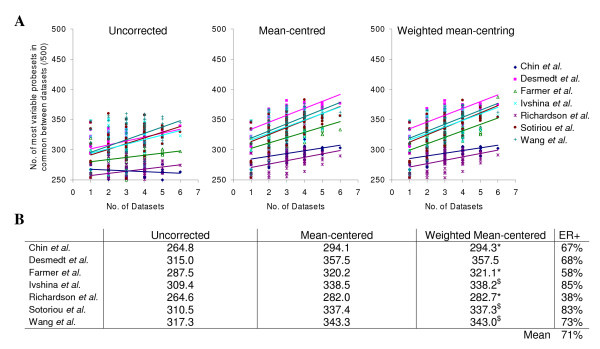
**Combining greater numbers of datasets leads to a greater overlap in differentially expressed probesets**. Lists of the five hundred probesets with the highest variance were generated for each dataset and combinations of up to six datasets and the number of probesets in common between these lists were plotted for each dataset. A, Plots show the number of common probesets between each individual dataset and other single or combined datasets. B, Overall mean numbers of genes in common for each dataset.

An alternative approach to assess whether mean-centering improves comparisons across published datasets was to identify lists of the five hundred most highly differentially expressed probesets across each dataset (those with the highest variance) and compare these gene lists with the most differentially expressed probesets from other single or combined datasets. Bringing together greater numbers of datasets or tumours increased the overlap in differentially expressed probesets (Figure 5). The number of probesets in common was significantly greater with mean-centering (p = 3.6 × 10^-107^) or weighted mean-centering (p = 9.2 × 10^-106^) over uncorrected data, although there was no significant improvement between the two methods (p = 0.5). The mean number of genes in common was higher following weighted mean-centering than mean-centering when the dataset was made up of less ER-positive tumours (Chin *et al.*, Farmer *et al*. and Richardson *et al*.) and lower when the dataset was made up of more ER-positive tumours (Ivshina *et al*., Sotoriou *et al*., Wang *et al.*).

## Discussion

Mean-centering has been widely used in the past to compare relative gene expression of high and lowly expressed genes together within a single dataset, particularly for heatmaps and clustering programs [35]. However, this is the first study to assess the utility of mean-centering for minimising the effects of dataset-specific bias and integration of multiple datasets. An unknown, systematic, multiplicative bias associated with each group of arrays processed together ('dataset') is simply removed when the GeneChips are considered relative to each other. The approach clearly shows significant improvements in the degree of correspondence found across datasets, without any loss of internal coherence within each of the initial datasets from which the integrated dataset is assembled. Relative intensities within each individual dataset are left unchanged (Figure 1D), with the consequence that both fold-changes and p-values produced by techniques such as SAM, remain identical to those found prior to correction (Table 1). Therefore, balanced corrected datasets can be treated with at least as much confidence as the initial uncorrected data. We have also demonstrated that combining greater numbers of datasets or tumours increases the overlap in differentially expressed probesets between studies and that this is further improved with mean-centering.

A number of previous studies have also investigated the level of consensus found between different experimental datasets. The mean-centering approach out-performed a distance weighted discrimination method [34] that attempted to adjust for systematic microarray data biases for integrating breast cancer tumour microarray data generated on different platforms. This group stated that they had also applied this technique to 'merge two distinct Affymetrix breast tumor datasets together' and 'saw similar, but less dramatic results due to fewer systematic biases present in datasets performed on the same Affymetrix microarrays' [34]. Our results suggest that there are many sources of systematic biases in Affymetrix data, which are highly significant and multiplicative, but that these can be largely corrected for, allowing the integration of datasets. An empirical Bayes method to adjust for batch effects [36] (ComBat; ) has also been used to integrate published datasets for meta-analysis [37]. This approach generated plots analogous to those in Additional File 8 for mean-centering and weighted mean-centering when ER status was used as a covariate (data not shown). The mean-centering method described in this study was used in a recent meta-analysis whilst our manuscript was under review [38], although no attempt was made to account for differences in dataset composition.

Combining two published studies without mean-centering, clearly demonstrated how dataset-specific biases can mask the biological differences between breast cancer tumour subtypes (Figure 2). The Farmer *et al. *[11] dataset was generated from trucut tumour biopsies (4 × 2.5 μm sections), necessitating RNA amplification prior to hybridization to U133A GeneChips. By contrast, RNA in the Richardson *et al. *2006 study [10] was derived from tumours following surgical removal, so amplification was not required prior to hybridisation to U133 plus 2.0 GeneChips. Despite the experimental differences between the studies, both of which have been shown above to lead to significant deviations in measured raw intensities in our validation datasets, mean-centering appears to reconcile the data and leads to the identification of biologically plausible relationships not found when combining uncorrected data.

The gold standard for demonstrating the power of a gene expression classifier is to test it against independent datasets. However, if the molecular profile of a set of tumours is representative of its patient characteristics, then any prognostic signature will be dependant upon the composition of the patient cohort and therefore be dataset-specific. Thus in order to generate accurate prognostic predictions, the characteristics of this second 'test' dataset must have similar characteristics to the first 'training' set [22]. Recently, strong time dependence was identified for a prognostic signature when comparing an independent validation dataset with a longer median follow-up time (14 years) compared to the original study (8 years) [17]. A number of recent microarray studies have been performed after increasing the size of a dataset with additional samples [8,18,20,33], however it is unclear whether subsequent changes in the results are due to changes in the sample composition of the extended dataset or simply to technical effects arising from the microarrays being processed in different batches. Some studies have also based their findings upon combined data from more than one type of Affymetrix GeneChip without evaluating any GeneChip-specific effects.

By integrating six published datasets with patient follow-up information we have demonstrated that combining breast cancer datasets can increase the accuracy of prognosis prediction and that this can be improved by removing systematic, multiplicative bias. The most accurate prognosis predictions are generated when the test-sets closely share the patient and tumour characteristics of the training-sets. An alternative approach to building ever larger combined datasets representing the whole breast cancer population, would be to concentrate on generating gene expression classifiers for clearly defined groups of patients (e.g. node-negative, ER-positive from patients aged 50–60, with 10 years of follow-up). Strict entry criterion would severely restrict the number of tumours eligible for inclusion, whilst taking no account of possible unknown confounding factors. In clinical practice, we urgently need single sample predictors [14], applicable to all patients and our work strongly suggests that these will be best generated from the largest possible cohorts (or integrated datasets) representing the wider population, which will involve large international collaborations and public sharing of data. The current consensus for best practices for breast cancer treatment are based upon bringing together data for hundreds of trials representing thousands of women within the Early Breast Cancer Trialists' Collaborative Group (EBCTCG). If we can begin to bring together many large datasets of gene expression data free from dataset-specific bias the opportunity exists to create a highly valuable resource. A possible 'new' breast cancer subtype characterized by the high expression of interferon regulated genes [14] was identified by cluster analysis of a combined (non-Affymetrix) dataset of 315 breast tumours, which is consistent with the notion that rare molecular subtypes will only be detected with larger datasets. Our findings are in agreement with the conclusions of a recent study [39] that integrated breast tumour datasets generated on two different microarray platforms; they also showed that the gene expression profile generated by integrated analysis of multiple datasets achieves better prediction of breast cancer recurrence, and that the performance of profiles is confounded by the known and unknown clinical background of patients [39]. In the current study however, we demonstrate improved prediction of prognosis in datasets derived from the *same *platform integrated using a simpler scaling method of the raw data rather than a normalisation method reliant on fold changes. One limitation of our study is that it was not possible to use a single definition of follow-up endpoint across the published datasets, in each case we used the most conservative indicator of relapse available (recurrence-free survival, disease-free survival and distant metastasis-free survival) rather than overall survival. There was also some variation in both patient age and tumour size between the studies (Table 3). Variation in gene expression due to the heterogeneity of patient characteristics has begun to be addressed by studies that investigate the effects of age [40], race [41,42] and differences in risk factors [43,44] for breast cancer. Integrating large breast tumour gene expression datasets will potentially enable us to uncover more subtle population-level associations, providing that all clinical details and follow-up information is consistent, complete and made publicly available.

## Conclusion

Systematic multiplicative biases are introduced at many stages of microarray experiments, however they can easily be accounted for, which can enable raw data to be directly integrated from different gene expression datasets in order to generate results with improved statistical power and greater biological significance.

## Methods

### RNA preparation

Generation and processing of RNA comparing the two breast cell lines, MCF7 (cancer) and MCF10A (normal immortalised mammary epithelial) was described previously [45,46]. Briefly, cells were grown in Dulbecco's modified Eagle's medium (DMEM) with 10% fetal calf serum (MCF7) or DMEM/F12 with 5% horse serum, 2 ng/ml, 0.5 μg/ml hydrocortisone, 0.5 μg/ml cholera toxin, and 5 μg/ml insulin (MCF10A). Minimally passaged cells (< 20) were obtained from the American Type Culture Collection ATCC. RNA was isolated using Trizol^® ^(Ambion) according to manufacturer's instructions, purified using Qiagen RNeasy columns (Qiagen, Valencia, CA) and quantified using a Nanodrop spectrophotometer (Labtech). The quality and amount of starting RNA was confirmed with an Agilent Bioanalyzer 2100 (Agilent) prior to labelling and hybridisation to HG-U133A, HG-U133 plus 2.0, or Human Exon 1.0ST GeneChips (Affymetrix) using either the Affymetrix standard or small sample preparation protocols as previously described [45].

### GeneChip processing and analysis

Each experiment was repeated in triplicate, with three samples per cell line for each amount of starting total RNA, protocol or GeneChip used (1 dataset = 3 × MCF7 and 3 × MCF10A = 6 GeneChips). The HGU133A Genechips for the standard protocol and amplification experiments were scanned using a GeneArray 2500 scanner and the HG-U133 plus 2.0, and Exon 1.0ST Genechips were scanned using a GeneChip Scanner 3000. All MCF7 and MCF10A microarray data is MIAME compliant and accessible via MIAME VICE [47]. All protocols are described in full here, . Raw spot readings were processed using R [31] and Bionconductor [48]. Probeset summarisation was done using MAS 5.0 and RMA [49] as implemented in the Simpleaffy package [50] or plier algorithm from Affymetrix ExACT software. Mappings between the Exon and U133A plus 2.0 GeneChips were performed as described previously [46]. Alternative cell description files, relating probesets to unigene sequences were implemented as described previously [5]. Lists of common significantly differentially expressed genes before and after mean batch-centering were identified using SAM [51] analysis (siggenes BioConductor package) by adjust Δ value to find the top 1000 differentially expressed probesets using each protocol, as described previously [46].

### Correction by batch mean-centering

The mean expression level per probeset for a given dataset is subtracted from the individual GeneChip expression level on the Log2 scale. This can simply be performed in R using the '*rowMeans*' function. Whilst preparing the manuscript we also noticed that this can be achieved using the '*pamr.batchadjust*' function within the pamr Bioconductor package.

### Published data

Affymetrix data was downloaded from a total of ten datasets from published studies listed in Table 2 from the Gene Expression Omnibus [3] or Array Express [1] repositories. Raw .cel files were not available for the Wang *et al. *dataset, so all other datasets were normalized as in this study using the MAS 5.0 algorithm with a target intensity of 600 as implemented in the Simpleaffy package [50], using R [31] within BioConductor [48]. NetAffx [9] was used to identify Affymetrix probesets representing the 'intrinsic gene set' previously used to classify human breast tumours [8]. Centered average linkage clustering was performed using the Cluster [35] and TreeView programs as described previously [7]. Supervised principal components analysis using the Superpc for R package was used as previously described [29,30], in order to compare the predictive power of combining different published datasets. The follow up endpoints for the Loi *et al.*, Pawitan *et al. *and Sotoriou *et al. *datasets were recurrence-free survival, for Desmedt *et al. *and Ivshina *et al. *datasets it was disease-free survival and for the Minn *et al. *and Wang *et al. *datasets it was distant metastasis-free survival.

## Competing interests

The authors declare that they have no competing interests.

## Authors' contributions

AHS conceived and designed the study. RBC, YH and SDP processed the lab experiments. AHS, GS and MJO performed the computational experiments and analysis. AHS, CJM, AH and RBC drafted the manuscript. All authors read and approved the final manuscript.

## Pre-publication history

The pre-publication history for this paper can be accessed here:



## Supplementary Material

Additional file 1 **Comparison of Affymetrix gene expression data generated using different generations of GeneChips, scanning hardware and protocols**. A, Comparing the fold change between replicates across datasets is clearly impractical (grey). However, following mean batch-centering there is good correlation (black). B, Comparison of mean raw expression levels for amplified and unamplified MCF10A replicates before (grey) and after mean batch-centering (black). C, Overall transcriptome similarity of individual GeneChips demonstrated with Pearson clustering. D, Fold changes are unaffected by mean batch-centering. Click here for file

Additional file 2 **Concordance of mean expression values of data generated from different experiments**. Pearson correlation coefficients are given for uncorrected and mean batch-corrected data, for RMA and MAS5 data, and using alternative cdf files [5]. Click here for file

Additional file 3 **The top 50 differentially expressed probesets between basal and non basal-like/luminal tumours were identified across datasets.** Those probesets in common are listed. Before: comparison was performed prior to mean batch-centering. After: comparison was performed following mean batch-centering. Click here for file

Additional file 4 **Summary of the effect of mean batch-centering on data generated from published studies.** Lists of the top 50 differentially expressed probesets between basal and non basal-like/luminal tumours were identified within and across datasets, before and after mean batch-centering. SAM Common: for each column two different pairwise comparisons using SAM were performed, and the top 50 probesets identified for each comparison. The number reported is the intersection between two lists. UC = uncorrected. MC = Mean centering correction. Click here for file

Additional file 5 **Examples of cross-validation and survival curves from supervised principal components analysis**. Cross validation plots (A, C) and Kaplan Meir recurrence curves (B, C) using the Wang *et al. *dataset as the test set and either a single (Pawitan *et al*.) dataset (A, B) or five (Chin *et al*., Desmedt *et al.*, Ivshina *et al*., Pawitan *et al. *and Sotoriou *et al.*) datasets (C, D) combined as the training set. Values at the top of the cross validation plots are the numbers of probesets used to create the profiles; the black, red and green lines represent the 1^st^, 2^nd ^and 3^rd ^principal components respectively. Click here for file

Additional file 6 **Full matrix of the 1109 *R*^2 ^and *p-values *for all possible combinations of the six training and test sets**. The *R*^2 ^statistic (Cox proportional hazards model) measures the percentage of the variation in time to recurrence that is explained by each combination of test datasets. The *p-values *are the associated log-rank statistic obtained when applying the test dataset to the training dataset. Click here for file

Additional file 7 **Comparison of published datasets composed of different ratios of basal and luminal tumours**. The number of basal (red) and luminal (blue) tumours from The Farmer *et al. *(*italics*) and Richardson *et al. *studies was varied in order to compare the effect of dataset composition, between (A, B, C) and across (D, E, F) the studies. The datasets were either uncorrected (light grey dots), mean-centered (black open squares) or weighted mean-centered (dark grey open circles). UC = uncorrected, MC = mean-centered. Click here for file

Additional file 8 **Effects of combining datasets composed solely of ER+ or ER- breast tumours**. Datasets from Loi *et al. *[32] and Minn *et al. *[33] that are composed wholly of ER+ or ER- tumours have distorted levels of ESR1 transcript if integrated with datasets composed of both ER+ and ER- tumours. Replacing any of the six heterogenous datasets with homogeneous datasets results in a dramatic reduction in the correlation between dataset or tumour number and the association with principal components and recurrence (B). Click here for file

Additional file 9 **Weighted-mean centering does not significantly improve prognostic prediction when combining datasets or tumours of mean-centering**. Five datasets with recorded ER status from immunohistochemistry were used to assess the correction methods as in Figure 4. The *R*^2 ^statistic (Cox proportional hazards model) is an assessment of the performance of the predictor generated using each combination of training datasets and the remaining test datasets, generated using supervised principal components analysis. Median values are used where a training dataset was used to asses more than one test dataset (up to 5). *R*^2 ^and *p-value *results for all possible combinations of training datasets and test datasets (1016) are given in the matrix in Additional Table 5. Click here for file

Additional file 10 **Distance-weighted discrimination (DWD) method**. Comparison of the DWD method (green dots) between (A, B) and across (C, D) validation (A, C) and published (B, D) datasets with mean-(red dots) and weighted mean-(blue circles) centering (see Table 3 for SAM analysis). E, DWD correction of the two breast tumour gene expression profiles generated by the two published studies as in Figure 2. Clustering of tumours based upon 640 probesets representing Sorlie *et al. *[8] 'intrinsic' genes. Thumbnail shows all 640 probesets. i) Tumours classified by Richardson *et al. *[10] red = basal-like, blue = non-basal like, pink = BRCA1; tumours classified by Farmer *et al. *[11] red = basal, blue = luminal, green = apocrine. Clusters of genes associated with the 'Sorlie subtypes' are highlighted as follows; ii) ERBB2 gene cluster, iii) luminal A gene cluster, iv) basal gene cluster. v) Centroid prediction was used to assign the tumours to the five Norway/Stanford subtypes – basal (red), luminal A (dark blue), luminal B (light blue), ERBB2 (purple) and normal-like (green), unassigned (grey). Click here for file

## References

[B1] Brazma A, Kapushesky M, Parkinson H, Sarkans U, Shojatalab M (2006). Data storage and analysis in ArrayExpress. Methods Enzymol.

[B2] Chu TM, Deng S, Wolfinger R, Paules RS, Hamadeh HK (2004). Cross-site comparison of gene expression data reveals high similarity. Environ Health Perspect.

[B3] Barrett T, Suzek TO, Troup DB, Wilhite SE, Ngau WC, Ledoux P, Rudnev D, Lash AE, Fujibuchi W, Edgar R (2005). NCBI GEO: mining millions of expression profiles – database and tools. Nucleic Acids Res.

[B4] Pepper SD, Saunders EK, Edwards LE, Wilson CL, Miller CJ (2007). The utility of MAS5 expression summary and detection call algorithms. BMC Bioinformatics.

[B5] Dai M, Wang P, Boyd AD, Kostov G, Athey B, Jones EG, Bunney WE, Myers RM, Speed TP, Akil H (2005). Evolving gene/transcript definitions significantly alter the interpretation of GeneChip data. Nucleic Acids Res.

[B6] Perou CM, Sorlie T, Eisen MB, Rijn M van de, Jeffrey SS, Rees CA, Pollack JR, Ross DT, Johnsen H, Akslen LA (2000). Molecular portraits of human breast tumours. Nature.

[B7] Sorlie T, Perou CM, Tibshirani R, Aas T, Geisler S, Johnsen H, Hastie T, Eisen MB, Rijn M van de, Jeffrey SS (2001). Gene expression patterns of breast carcinomas distinguish tumor subclasses with clinical implications. Proc Natl Acad Sci USA.

[B8] Sorlie T, Tibshirani R, Parker J, Hastie T, Marron JS, Nobel A, Deng S, Johnsen H, Pesich R, Geisler S (2003). Repeated observation of breast tumor subtypes in independent gene expression data sets. Proc Natl Acad Sci USA.

[B9] Liu G, Loraine AE, Shigeta R, Cline M, Cheng J, Valmeekam V, Sun S, Kulp D, Siani-Rose MA (2003). NetAffx: Affymetrix probesets and annotations. Nucleic Acids Res.

[B10] Richardson AL, Wang ZC, De Nicolo A, Lu X, Brown M, Miron A, Liao X, Iglehart JD, Livingston DM, Ganesan S (2006). X chromosomal abnormalities in basal-like human breast cancer. Cancer Cell.

[B11] Farmer P, Bonnefoi H, Becette V, Tubiana-Hulin M, Fumoleau P, Larsimont D, Macgrogan G, Bergh J, Cameron D, Goldstein D (2005). Identification of molecular apocrine breast tumours by microarray analysis. Oncogene.

[B12] Calza S, Hall P, Auer G, Bjohle J, Klaar S, Kronenwett U, Liu ET, Miller L, Ploner A, Smeds J (2006). Intrinsic molecular signature of breast cancer in a population-based cohort of 412 patients. Breast Cancer Res.

[B13] Sorlie T, Wang Y, Xiao C, Johnsen H, Naume B, Samaha RR, Borresen-Dale AL (2006). Distinct molecular mechanisms underlying clinically relevant subtypes of breast cancer: Gene expression analyses across three different platforms. BMC Genomics.

[B14] Hu Z, Fan C, Oh DS, Marron JS, He X, Qaqish BF, Livasy C, Carey LA, Reynolds E, Dressler L (2006). The molecular portraits of breast tumors are conserved across microarray platforms. BMC Genomics.

[B15] Kouros-Mehr H, Slorach EM, Sternlicht MD, Werb Z (2006). GATA-3 maintains the differentiation of the luminal cell fate in the mammary gland. Cell.

[B16] Chin K, DeVries S, Fridlyand J, Spellman PT, Roydasgupta R, Kuo WL, Lapuk A, Neve RM, Qian Z, Ryder T (2006). Genomic and transcriptional aberrations linked to breast cancer pathophysiologies. Cancer Cell.

[B17] Desmedt C, Piette F, Loi S, Wang Y, Lallemand F, Haibe-Kains B, Viale G, Delorenzi M, Zhang Y, d'Assignies MS (2007). Strong time dependence of the 76-gene prognostic signature for node-negative breast cancer patients in the TRANSBIG multicenter independent validation series. Clin Cancer Res.

[B18] Ivshina AV, George J, Senko O, Mow B, Putti TC, Smeds J, Lindahl T, Pawitan Y, Hall P, Nordgren H (2006). Genetic reclassification of histologic grade delineates new clinical subtypes of breast cancer. Cancer Res.

[B19] Pawitan Y, Bjohle J, Amler L, Borg AL, Egyhazi S, Hall P, Han X, Holmberg L, Huang F, Klaar S (2005). Gene expression profiling spares early breast cancer patients from adjuvant therapy: derived and validated in two population-based cohorts. Breast Cancer Res.

[B20] Sotiriou C, Wirapati P, Loi S, Harris A, Fox S, Smeds J, Nordgren H, Farmer P, Praz V, Haibe-Kains B (2006). Gene expression profiling in breast cancer: understanding the molecular basis of histologic grade to improve prognosis. J Natl Cancer Inst.

[B21] Wang Y, Klijn JG, Zhang Y, Sieuwerts AM, Look MP, Yang F, Talantov D, Timmermans M, Meijer-van Gelder ME, Yu J (2005). Gene-expression profiles to predict distant metastasis of lymph-node-negative primary breast cancer. Lancet.

[B22] Sims AH, Ong KR, Clarke RB, Howell A (2006). High-throughput genomic technology in research and clinical management of breast cancer. Exploiting the potential of gene expression profiling: is it ready for the clinic?. Breast Cancer Res.

[B23] van 't Veer LJ, Dai H, Vijver MJ van de, He YD, Hart AA, Mao M, Peterse HL, Kooy K van der, Marton MJ, Witteveen AT (2002). Gene expression profiling predicts clinical outcome of breast cancer. Nature.

[B24] Adler AS, Lin M, Horlings H, Nuyten DS, Vijver MJ van de, Chang HY (2006). Genetic regulators of large-scale transcriptional signatures in cancer. Nat Genet.

[B25] Chang HY, Nuyten DS, Sneddon JB, Hastie T, Tibshirani R, Sorlie T, Dai H, He YD, Van't Veer LJ, Bartelink H (2005). Robustness, scalability, and integration of a wound-response gene expression signature in predicting breast cancer survival. Proc Natl Acad Sci USA.

[B26] Chi JT, Wang Z, Nuyten DS, Rodriguez EH, Schaner ME, Salim A, Wang Y, Kristensen GB, Helland A, Borresen-Dale AL (2006). Gene expression programs in response to hypoxia: cell type specificity and prognostic significance in human cancers. PLoS Med.

[B27] Liu R, Wang X, Chen GY, Dalerba P, Gurney A, Hoey T, Sherlock G, Lewicki J, Shedden K, Clarke MF (2007). The prognostic role of a gene signature from tumorigenic breast-cancer cells. N Engl J Med.

[B28] Fan C, Oh DS, Wessels L, Weigelt B, Nuyten DS, Nobel AB, van't Veer LJ, Perou CM (2006). Concordance among gene-expression-based predictors for breast cancer. N Engl J Med.

[B29] Bair E, Hastie T, Debashis P, Tibshirani R (2004). Prediction by supervised principal components. Stanford Tech Report.

[B30] Bair E, Tibshirani R (2004). Semi-supervised methods to predict patient survival from gene expression data. PLoS Biol.

[B31] Ihaka R, Gentleman R (1996). R: a language for data analysis and graphics. Journal of Computational and Graphical Statistics.

[B32] Loi S, Haibe-Kains B, Desmedt C, Lallemand F, Tutt AM, Gillet C, Ellis P, Harris A, Bergh J, Foekens JA (2007). Definition of clinically distinct molecular subtypes in estrogen receptor-positive breast carcinomas through genomic grade. J Clin Oncol.

[B33] Minn AJ, Gupta GP, Padua D, Bos P, Nguyen DX, Nuyten D, Kreike B, Zhang Y, Wang Y, Ishwaran H (2007). Lung metastasis genes couple breast tumor size and metastatic spread. Proc Natl Acad Sci USA.

[B34] Benito M, Parker J, Du Q, Wu J, Xiang D, Perou CM, Marron JS (2004). Adjustment of systematic microarray data biases. Bioinformatics.

[B35] Eisen MB, Spellman PT, Brown PO, Botstein D (1998). Cluster analysis and display of genome-wide expression patterns. Proc Natl Acad Sci USA.

[B36] Johnson WE, Li C, Rabinovic A (2007). Adjusting batch effects in microarray expression data using empirical Bayes methods. Biostatistics.

[B37] Acharya CR, Hsu DS, Anders CK, Anguiano A, Salter KH, Walters KS, Redman RC, Tuchman SA, Moylan CA, Mukherjee S (2008). Gene expression signatures, clinicopathological features, and individualized therapy in breast cancer. Jama.

[B38] Ben-Porath I, Thomson MW, Carey VJ, Ge R, Bell GW, Regev A, Weinberg RA (2008). An embryonic stem cell-like gene expression signature in poorly differentiated aggressive human tumors. Nat Genet.

[B39] Zhang Z, Chen D, Fenstermacher DA (2007). Integrated analysis of independent gene expression microarray datasets improves the predictability of breast cancer outcome. BMC Genomics.

[B40] Yau C, Fedele V, Roydasgupta R, Fridlyand J, Hubbard A, Gray JW, Chew K, Dairkee SH, Moore DH, Schittulli F (2007). Aging impacts transcriptome but not genome of hormone-dependent breast cancers. Breast Cancer Res.

[B41] Amend K, Hicks D, Ambrosone CB (2006). Breast cancer in african-american women: differences in tumor biology from European-american women. Cancer Res.

[B42] Carey LA, Perou CM, Livasy CA, Dressler LG, Cowan D, Conway K, Karaca G, Troester MA, Tse CK, Edmiston S (2006). Race, breast cancer subtypes, and survival in the Carolina Breast Cancer Study. Jama.

[B43] Millikan RC, Newman B, Tse CK, Moorman PG, Conway K, Smith LV, Labbok MH, Geradts J, Bensen JT, Jackson S (2007). Epidemiology of basal-like breast cancer. Breast Cancer Res Treat.

[B44] Yang XR, Sherman ME, Rimm DL, Lissowska J, Brinton LA, Peplonska B, Hewitt SM, Anderson WF, Szeszenia-Dabrowska N, Bardin-Mikolajczak A (2007). Differences in risk factors for breast cancer molecular subtypes in a population-based study. Cancer Epidemiol Biomarkers Prev.

[B45] Wilson CL, Pepper SD, Hey Y, Miller CJ (2004). Amplification protocols introduce systematic but reproducible errors into gene expression studies. Biotechniques.

[B46] Okoniewski MJ, Hey Y, Pepper SD, Miller CJ (2007). High correspondance between Affymetrix exon and standard expression arrays. Biotechniques.

[B47] MIAME VICE. http://bioinformatics.picr.man.ac.uk/vice.

[B48] Gentleman RC, Carey VJ, Bates DM, Bolstad B, Dettling M, Dudoit S, Ellis B, Gautier L, Ge Y, Gentry J (2004). Bioconductor: open software development for computational biology and bioinformatics. Genome Biol.

[B49] Irizarry RA, Hobbs B, Collin F, Beazer-Barclay YD, Antonellis KJ, Scherf U, Speed TP (2003). Exploration, normalization, and summaries of high density oligonucleotide array probe level data. Biostatistics.

[B50] Wilson CL, Miller CJ (2005). Simpleaffy: a BioConductor package for Affymetrix Quality Control and data analysis. Bioinformatics.

[B51] Tusher VG, Tibshirani R, Chu G (2001). Significance analysis of microarrays applied to the ionizing radiation response. Proc Natl Acad Sci USA.

[B52] Bergamaschi A, Kim YH, Wang P, Sorlie T, Hernandez-Boussard T, Lonning PE, Tibshirani R, Borresen-Dale AL, Pollack JR (2006). Distinct patterns of DNA copy number alteration are associated with different clinicopathological features and gene-expression subtypes of breast cancer. Genes Chromosomes Cancer.

