# Supplementary figures and images for: The removal of multiplicative, systematic bias allows integration of breast cancer gene expression datasets – improving meta-analysis and prediction of prognosis
Source: BMC Med Genomics. 2008 Sep 21;1:42. doi: 10.1186/1755-8794-1-42 (PMC2563019; doi:10.1186/1755-8794-1-42)

## Additional File 1.

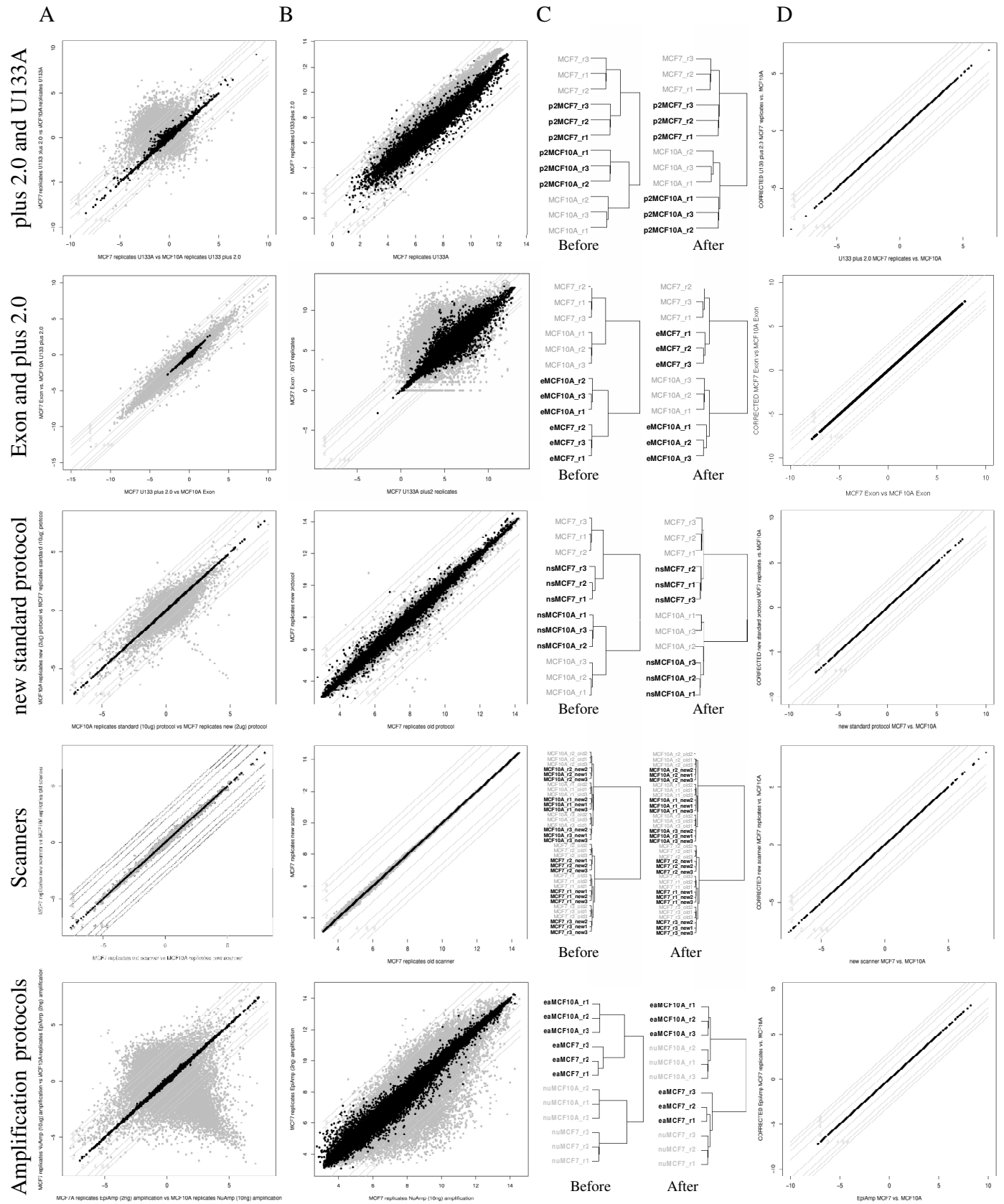

Supplement: Additional file 1 — Comparison of Affymetrix gene expression data generated using different generations of GeneChips, scanning hardware and protocols. A, Comparing the fold change between replicates across datasets is clearly impractical (grey). However, following mean batch-centering there is good correlation (black). B, Comparison of mean raw expression levels for amplified and unamplified MCF10A replicates before (grey) and after mean batch-centering (black). C, Overall transcriptome similarity of individual GeneChips demonstrated with Pearson clustering. D, Fold changes are unaffected by mean batch-centering. [file 1755-8794-1-42-S1.pdf]

## Additional File 5.

**A**

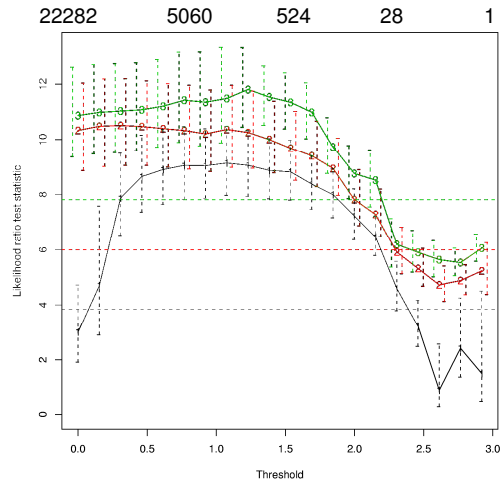

**B**

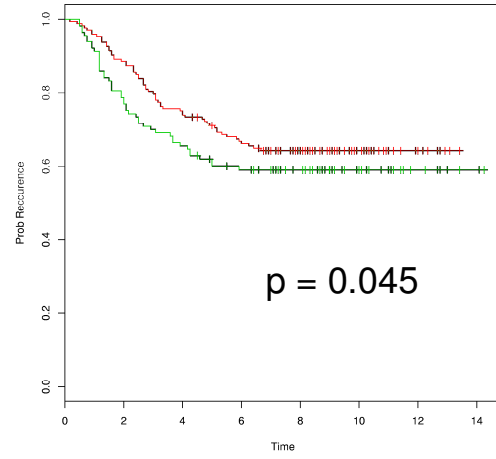

**C**

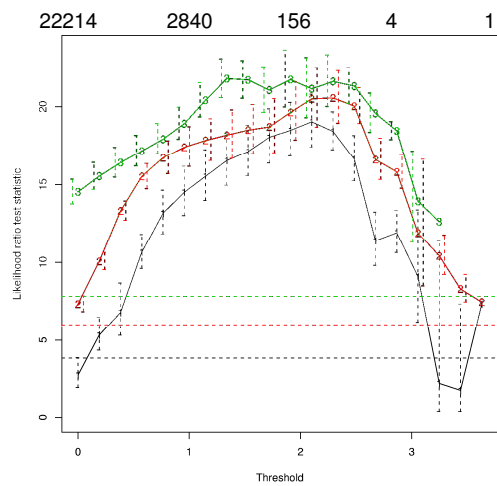

**D**

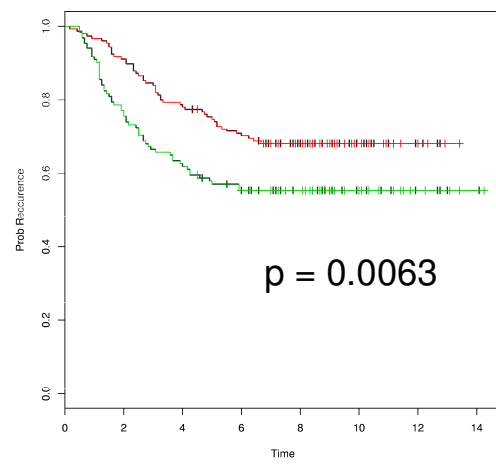

Supplement: Additional file 5 — Examples of cross-validation and survival curves from supervised principal components analysis. Cross validation plots (A, C) and Kaplan Meir recurrence curves (B, C) using the Wang et al. dataset as the test set and either a single (Pawitan et al.) dataset (A, B) or five (Chin et al., Desmedt et al., Ivshina et al., Pawitan et al. and Sotoriou et al.) datasets (C, D) combined as the training set. Values at the top of the cross validation plots are the numbers of probesets used to create the profiles; the black, red and green lines represent the 1st, 2nd and 3rd principal components respectively. [file 1755-8794-1-42-S5.pdf]

Additional File 7.

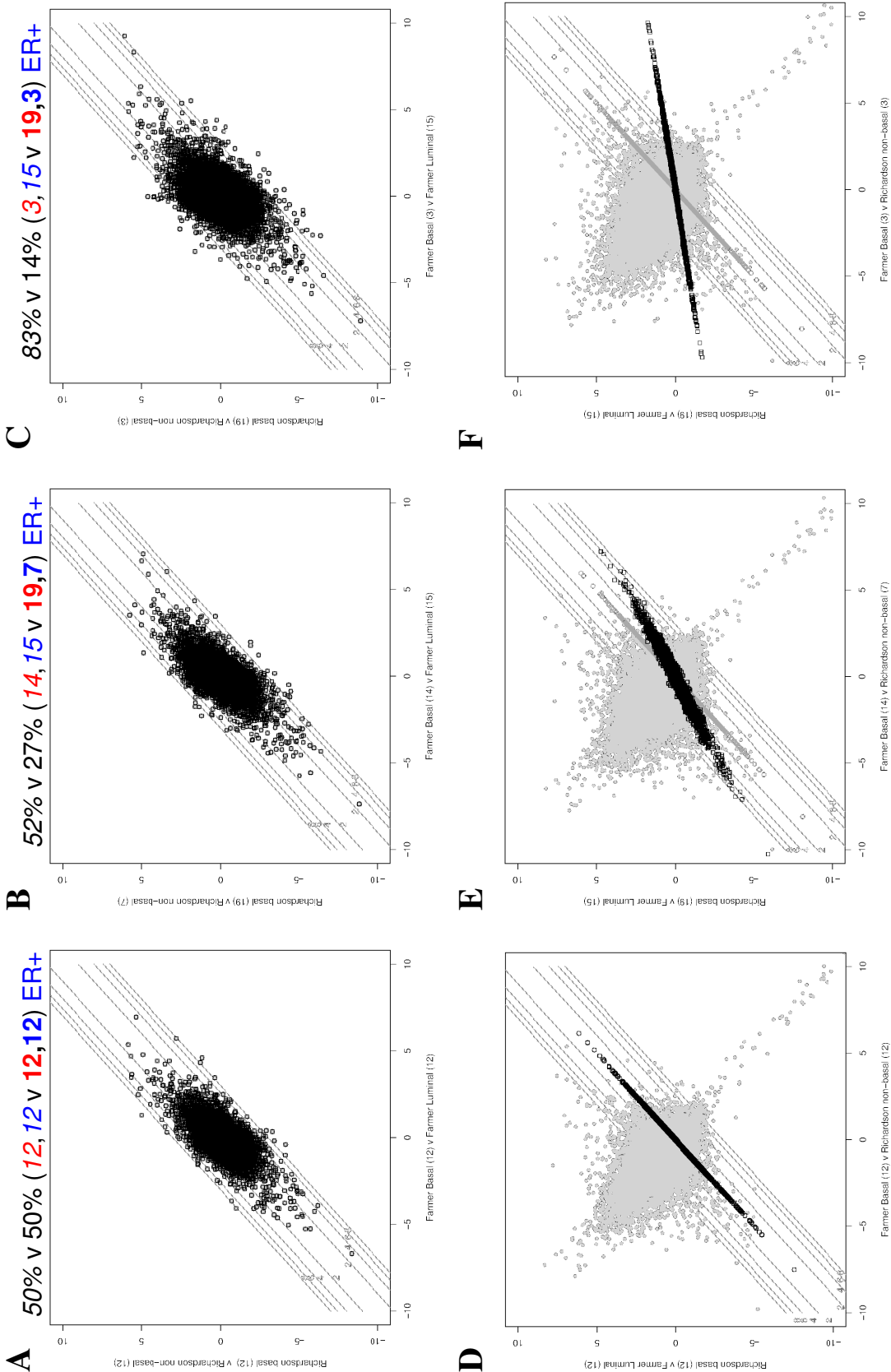

Supplement: Additional file 7 — Comparison of published datasets composed of different ratios of basal and luminal tumours. The number of basal (red) and luminal (blue) tumours from The Farmer et al. (italics) and Richardson et al. studies was varied in order to compare the effect of dataset composition, between (A, B, C) and across (D, E, F) the studies. The datasets were either uncorrected (light grey dots), mean-centered (black open squares) or weighted mean-centered (dark grey open circles). UC = uncorrected, MC = mean-centered. [file 1755-8794-1-42-S7.pdf]

## Additional File 8

**A**

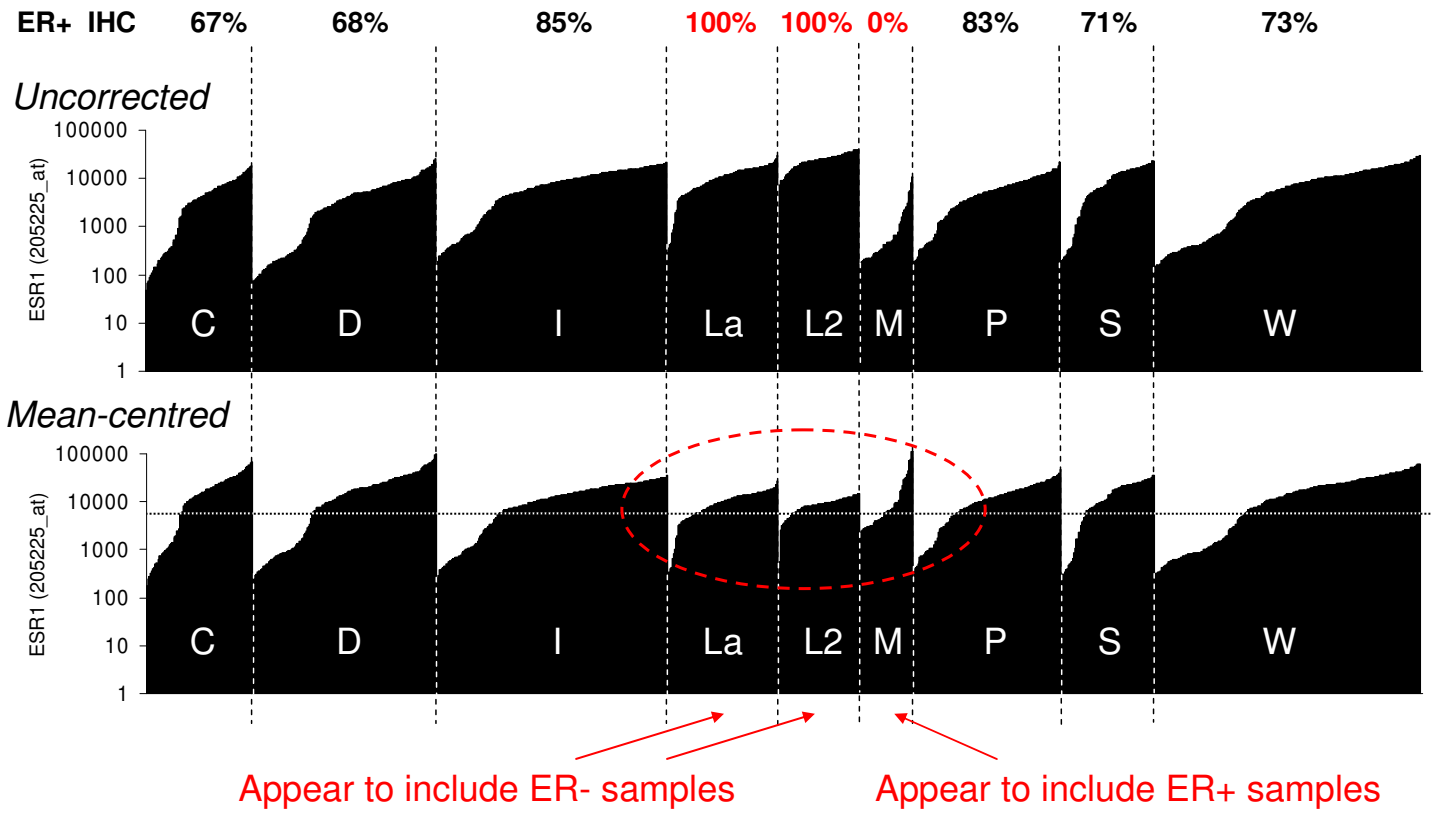

**B**

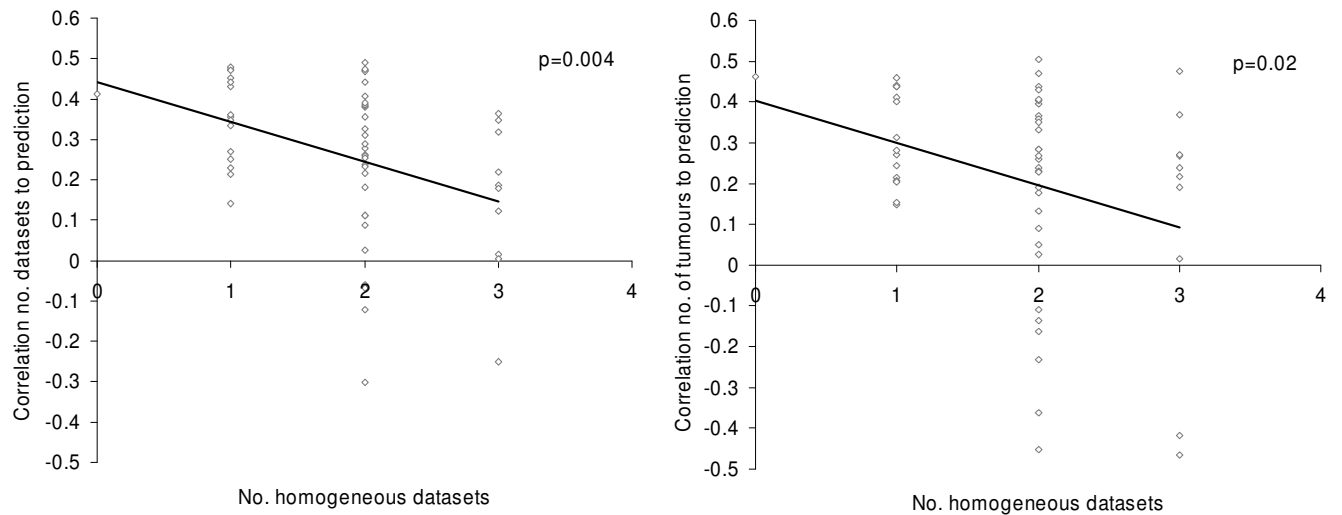

Supplement: Additional file 8 — Effects of combining datasets composed solely of ER+ or ER- breast tumours. Datasets from Loi et al. [32] and Minn et al. [33] that are composed wholly of ER+ or ER- tumours have distorted levels of ESR1 transcript if integrated with datasets composed of both ER+ and ER- tumours. Replacing any of the six heterogenous datasets with homogeneous datasets results in a dramatic reduction in the correlation between dataset or tumour number and the association with principal components and recurrence (B). [file 1755-8794-1-42-S8.pdf]

Additional File 9

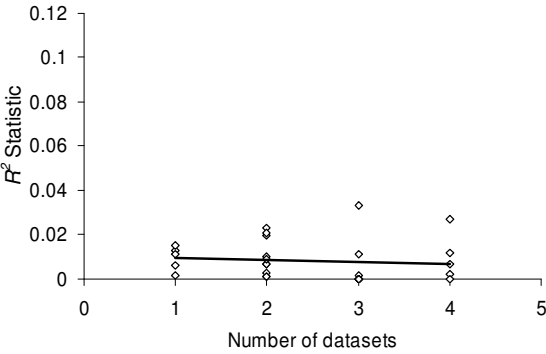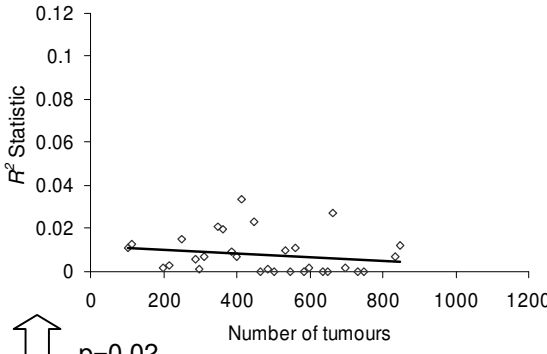

$p=0.02$

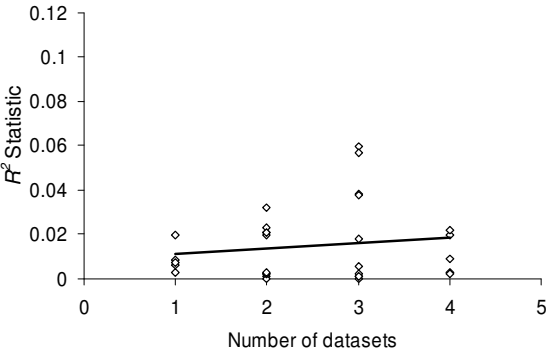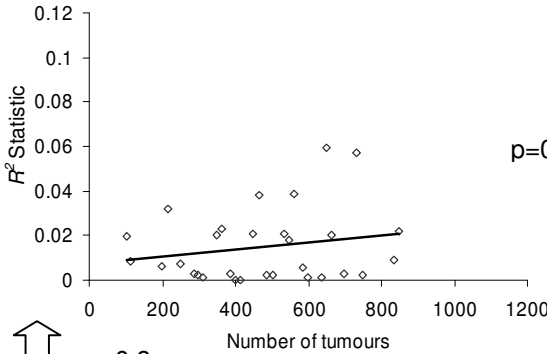

$p=0.00006$

$p=0.9$

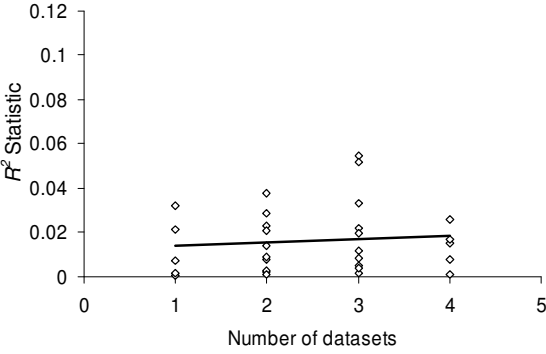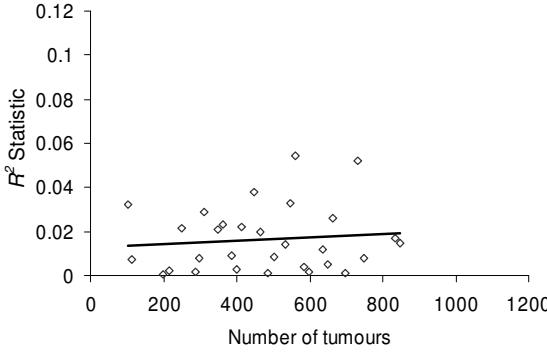

Supplement: Additional file 9 — Weighted-mean centering does not significantly improve prognostic prediction when combining datasets or tumours of mean-centering. Five datasets with recorded ER status from immunohistochemistry were used to assess the correction methods as in Figure 4. The R2 statistic (Cox proportional hazards model) is an assessment of the performance of the predictor generated using each combination of training datasets and the remaining test datasets, generated using supervised principal components analysis. Median values are used where a training dataset was used to asses more than one test dataset (up to 5). R2 and p-value results for all possible combinations of training datasets and test datasets (1016) are given in the matrix in Additional Table 5. [file 1755-8794-1-42-S9.pdf]

Additional File 10

A

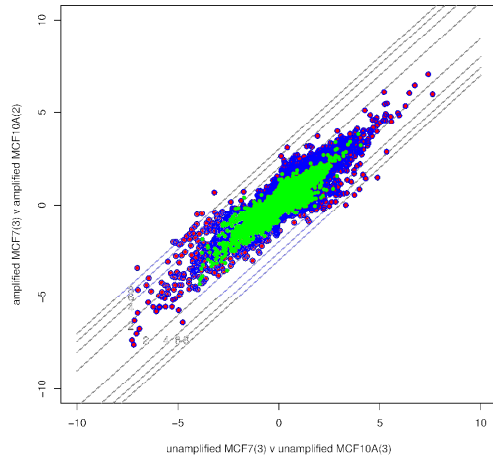

B

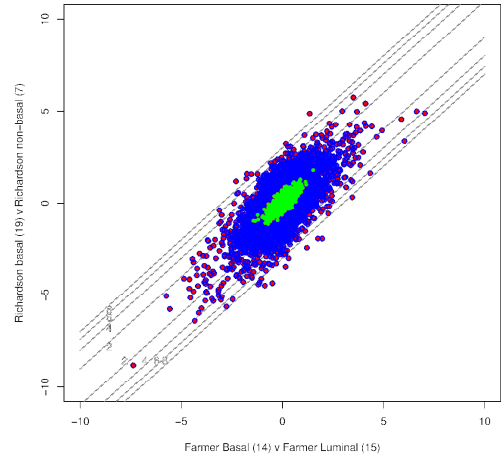

C

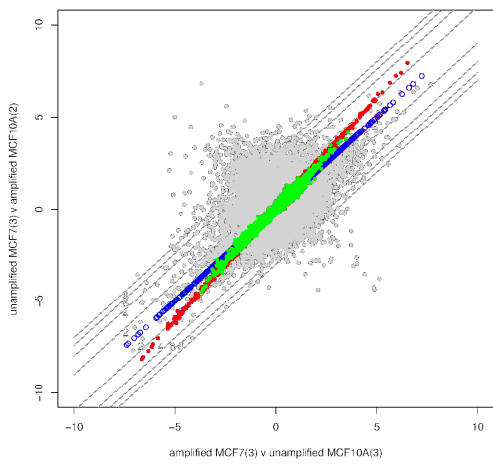

D

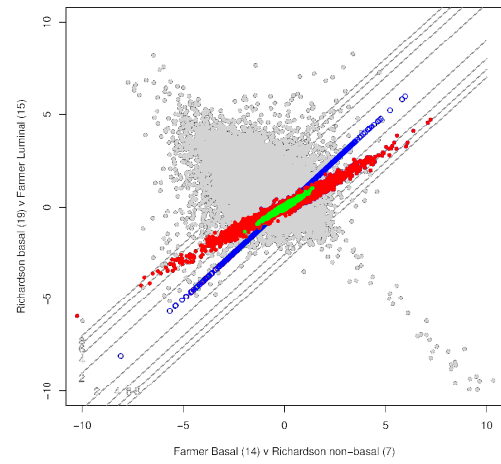

E

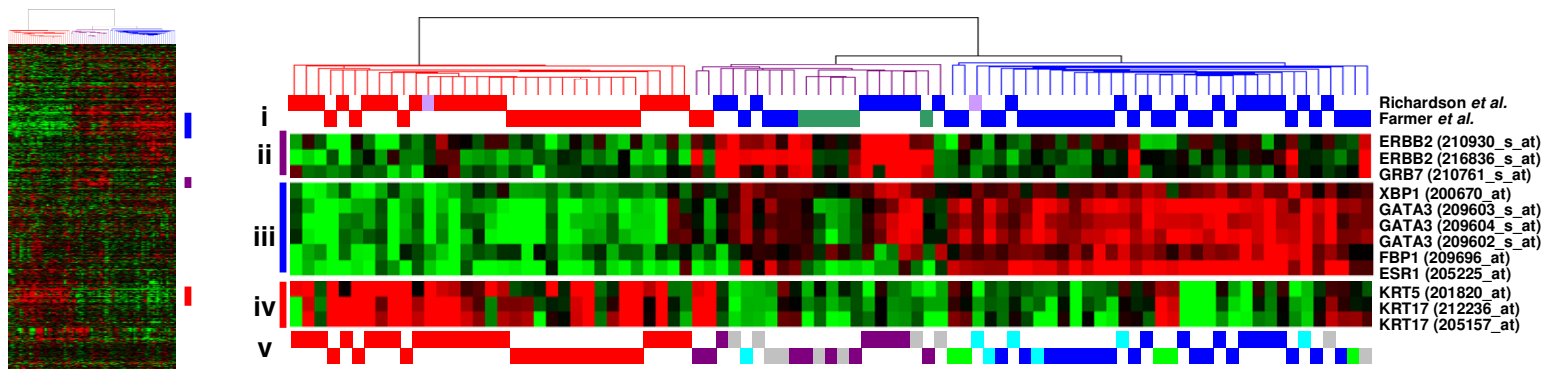

Supplement: Additional file 10 — Distance-weighted discrimination (DWD) method. Comparison of the DWD method (green dots) between (A, B) and across (C, D) validation (A, C) and published (B, D) datasets with mean-(red dots) and weighted mean-(blue circles) centering (see Table 3 for SAM analysis). E, DWD correction of the two breast tumour gene expression profiles generated by the two published studies as in Figure 2. Clustering of tumours based upon 640 probesets representing Sorlie et al. [8] 'intrinsic' genes. Thumbnail shows all 640 probesets. i) Tumours classified by Richardson et al. [10] red = basal-like, blue = non-basal like, pink = BRCA1; tumours classified by Farmer et al. [11] red = basal, blue = luminal, green = apocrine. Clusters of genes associated with the 'Sorlie subtypes' are highlighted as follows; ii) ERBB2 gene cluster, iii) luminal A gene cluster, iv) basal gene cluster. v) Centroid prediction was used to assign the tumours to the five Norway/Stanford subtypes – basal (red), luminal A (dark blue), luminal B (light blue), ERBB2 (purple) and normal-like (green), unassigned (grey). [file 1755-8794-1-42-S10.pdf]
