# Supplementary material for: The removal of multiplicative, systematic bias allows integration of breast cancer gene expression datasets – improving meta-analysis and prediction of prognosis
Source: BMC Med Genomics. 2008 Sep 21;1:42. doi: 10.1186/1755-8794-1-42 (PMC2563019; doi:10.1186/1755-8794-1-42)
Supplement: Additional file 2 — Concordance of mean expression values of data generated from different experiments. Pearson correlation coefficients are given for uncorrected and mean batch-corrected data, for RMA and MAS5 data, and using alternative cdf files [5]. [file 1755-8794-1-42-S2.pdf]

Additional File 2. Concordance of mean expression values of data generated from different experiments.

	Data from different validation experiments	Probesets	Before		After	
			MCF7	MCF10A	MCF7	MCF10A
Amplified and unamplified	Amplified 10ng and unamplified 10µg protocols, RMA, Affymetrix cdf summarisation (see Figure 1D)	22283	0.959	0.936	0.994	0.994
	Amplified 10ng and unamplified 10µg protocols, MAS5 (present ≥ 4/6 chips), Affymetrix cdf summarisation	9783	0.928	0.925	0.990	0.998
	Amplified 10ng and unamplified 10µg protocols, RMA, Unigene cdf summarisation	11707	0.956	0.933	0.994	0.993
	Amplified 10ng and unamplified 10µg protocols, MAS5 (present ≥ 4/6 chips), Unigene cdf summarisation	4729	0.901	0.819	0.987	0.985
Plus 2.0 and U133A	U133A and plus 2.0 arrays, RMA, common Affymetrix cdf summarisation probesets	22277	0.934	0.869	0.984	0.984
	U133A and plus 2.0 arrays, MAS5 (present ≥ 4/6 chips), common Affymetrix cdf summarisation probesets	11198	0.937	0.844	0.969	0.968
	U133A and plus 2.0 arrays, RMA, common Unigene cdf summarisation	11656	0.889	0.811	0.980	0.979
	U133A and plus 2.0 arrays, MAS5 (present ≥ 4/6 chips), common Unigene cdf summarisation	5740	0.840	0.725	0.962	0.958
Exon and plus 2.0	Exon v U133 plus 2 (consensus mapping) Plier and MAS5, Affymetrix cdf summarisation	44280	0.626	0.595	0.933	0.932
	Exon v U133 plus 2 (SIF mapping) Plier and MAS5, Affymetrix cdf summarisation	13730	0.591	0.559	0.946	0.947
New standard protocol	Standard 10µg and revised 2µg protocols, RMA, Affymetrix cdf summarisation	22283	0.983	0.977	0.996	0.996
	Standard 10µg and revised 2µg protocols, MAS5 (present ≥ 4/6 chips), Affymetrix cdf summarisation	10568	0.946	0.934	0.976	0.974
	Standard 10µg and revised 2µg protocols, RMA, Affymetrix cdf summarisation	10280	0.969	0.962	0.986	0.984
	Standard 10µg and revised 2µg protocols, MAS5, (present ≥ 4/6 chips), Unigene cdf summarisation	5175	0.937	0.922	0.972	0.969
Scanners	GeneChip 3000 and GeneArray 2500 scanners, RMA, Affymetrix cdf summarisation	22283	0.999	0.999	1.000	1.000
	GeneChip 3000 and GeneArray 2500 scanners, MAS5 (present ≥ 12/18 chips), Affymetrix cdf summarisation	9762	0.999	0.998	0.999	0.999
	GeneChip 3000 and GeneArray 2500 scanners, RMA, Unigene cdf summarisation	11707	0.999	0.999	1.000	1.000
	GeneChip 3000 and GeneArray 2500 scanners, MAS5 (present ≥ 12/18 chips), Unigene cdf summarisation	5588	0.999	0.998	0.999	0.999
Amplification protocols	NuGEN 10ng and EpiStem 2ng amplification, RMA, Affymetrix cdf summarisation	54675	0.893	0.893	0.993	0.993
	NuGEN 10ng and EpiStem 2ng amplification, MAS5 (present ≥ 4/6 chips), Affymetrix cdf summarisation	20958	0.821	0.828	0.990	0.992
	NuGEN 10ng and EpiStem 2ng amplification, RMA, Unigene cdf summarisation	11707	0.897	0.895	0.991	0.991
	NuGEN 10ng and EpiStem 2ng amplification, MAS5 (present ≥ 4/6 chips), Unigene cdf summarisation	95	0.710	0.533	0.795	0.643

Pearson correlation coefficients are given for uncorrected and mean batch-corrected data, for RMA and MAS5 data, and using alternative cdf files [5]
