# Supplementary material for: The removal of multiplicative, systematic bias allows integration of breast cancer gene expression datasets – improving meta-analysis and prediction of prognosis
Source: BMC Med Genomics. 2008 Sep 21;1:42. doi: 10.1186/1755-8794-1-42 (PMC2563019; doi:10.1186/1755-8794-1-42)
Supplement: Additional file 3 — The top 50 differentially expressed probesets between basal and non basal-like/luminal tumours were identified across datasets. Those probesets in common are listed. Before: comparison was performed prior to mean batch-centering. After: comparison was performed following mean batch-centering. [file 1755-8794-1-42-S3.pdf]

Additional File 3. The top 50 differentially expressed probesets between basal and non basal-like/ luminal tumours were identified across datasets.

SAM common (Richardson *et al.* 18 basal v Farmer *et al* 27 luminal AND Farmer *et al.* 16 basal v Richardson *et al.* 20 non basal-like)	
Before mean-centering	After mean-centering
ARFRP1 (215984_s_at)	unknown (215375_x_at)
ZFP36L2 (201369_s_at)	S100A1 (205334_at)
RPL27A (203034_s_at)	unknown (215686_x_at)
unknown (215182_x_at)	MFGE8 (210605_s_at)
PTMS (218045_x_at)	KIAA0182 (212056_at)
PTK7 (207011_s_at)	GABRP (205044_at)
ZFP36L2 (201367_s_at)	TFAP2B (214451_at)
FLOT2 (211299_s_at)	CHI3L2 (213060_s_at)
RPL32 (200674_s_at)	ESR1 (211234_x_at)
RAB14 (211503_s_at)	RAI17 (212124_at)
GNS (203676_at)	LAMA5 (210150_s_at)
PTK9 (214007_s_at)	C4A_///_C4B (208451_s_at_at)
GSTM4 (204149_s_at)	COL6A3 (201438_at)
STAT6 (201332_s_at)	CBR1 (209213_at)
NUMA1 (214251_s_at)	XBP1 (200670_at)
SENP3 (203871_at)	FABP7 (205030_at)
UBE1 (200964_at)	ERBB2 (216836_s_at)
PEA15 (200787_s_at)	CDC42EP4 (218063_s_at)
PTP4A2 (208615_s_at)	KIAA0310 (215696_s_at)
SENP3 (215113_s_at)	FZD7 (203705_s_at)
PPP1R15A (202014_at)	GSTP1 (200824_at)
PIP5K1A (210256_s_at)	GATA3 (209604_s_at)
PLXNB1 (215807_s_at)	TLE3 (212770_at)
PPP2CB (201374_x_at)	ASAH1 (213702_x_at)
HSF2 (209657_s_at)	VAV3 (218807_at)
AGXT (210327_s_at)	ESR1 (211235_s_at)
unknown (214008_at)	ESR1 (217190_x_at)
	VAV3 (218806_s_at)

Those probesets in common are listed. Before: comparison was performed prior to mean batch-centering. After: comparison was performed following mean batch-centering.
