# Supplementary material for: The removal of multiplicative, systematic bias allows integration of breast cancer gene expression datasets – improving meta-analysis and prediction of prognosis
Source: BMC Med Genomics. 2008 Sep 21;1:42. doi: 10.1186/1755-8794-1-42 (PMC2563019; doi:10.1186/1755-8794-1-42)
Supplement: Additional file 4 — Summary of the effect of mean batch-centering on data generated from published studies. Lists of the top 50 differentially expressed probesets between basal and non basal-like/luminal tumours were identified within and across datasets, before and after mean batch-centering. SAM Common: for each column two different pairwise comparisons using SAM were performed, and the top 50 probesets identified for each comparison. The number reported is the intersection between two lists. UC = uncorrected. MC = Mean centering correction. [file 1755-8794-1-42-S4.pdf]

Additional File 4. Summary of the effect of mean batch-centering on data generated from published studies.

	SAM Common			
	Richardson *et al*. 18 basal v Farmer *et al* 27 luminal (top 50)		Farmer *et al.* 16 basal v Richardson *et al*. 20 non basal-like (top 50)	
	UC	MC	UC	MC
Farmer *et al.* 16 basal v 27 luminal (top 50)	1	8	2	31
Richardson *et al.* 18 basal v 20 luminal (top 50)	2	6	4	26
Common to both Farmer *et al.* and Richardson *et al.* Luminal v Basal (total 13 probesets)	0	4	0	8

Lists of the top 50 differentially expressed probesets between basal and non basal-like/ luminal tumours were identified within and across datasets, before and after mean batch-centering. SAM Common: for each column two different pairwise comparisons using SAM were performed, and the top 50 probesets identified for each comparison. The number reported is the intersection between two lists. UC = uncorrected. MC = Mean centering correction.
